# Acellular dermal matrix combined with oxidized regenerated cellulose for partial breast reconstruction: Two case reports

**DOI:** 10.1097/MD.0000000000021217

**Published:** 2020-07-31

**Authors:** Jeeyeon Lee, Jung Dug Yang, Jeong Woo Lee, Junjie Li, Jin Hyang Jung, Wan Wook Kim, Chan Sub Park, Joon Seok Lee, Ho Yong Park

**Affiliations:** a Department of Surgery; b Department of Plastic Surgery, School of Medicine, Kyungpook National University; c Kyungpook National University Chilgok Hospital; d Kyungpook National University Hospital, Daegu, Korea; e Department of Surgery, Sichuan Province Cancer Hospital, Chengdu, China; f Joint Institute for Regenerative Medicine, Kyungpook National University, Daegu, Korea.

**Keywords:** acellular dermal matrix, breast, carcinoma, defect, oxidized regenerated cellulose

## Abstract

**Rationale:**

Filling materials for partial defect of the breast have rarely been developed because of safety and durability.

**Patient concerns:**

Two female patients (ages, 53 and 50 years) with breast cancer underwent partial mastectomy with sentinel lymph node biopsy.

**Diagnosis:**

Core needle biopsy revealed an invasive ductal carcinoma in both patients. Breast ultrasound showed hypoechoic nodules with irregular margins. Breast magnetic resonance imaging showed an irregularly shaped enhancing mass with duct extension in Patient 1 and irregularly shaped multifocal, enhancing masses with non-mass enhancement in Patient 2.

**Intervention:**

A combination method using acellular dermal matrix and oxidized regenerated cellulose was used for partial breast reconstruction. The safety and cosmetic outcomes were evaluated for both patients.

**Outcomes:**

There were no significant complications, and the breast shape and volume were well maintained, even 2 years after surgery. There was no postoperative tumor recurrence.

**Conclusion:**

The combination of acellular dermal matrix and oxidized regenerated cellulose for partial breast reconstruction can be a good option based on oncological safety and cosmetic outcome.

## 1 Introduction

Oncoplastic surgery refers to breast-conserving surgery with sufficient safety margins that consequently result in a naturally shaped breast.^[1]^ Although additional flap surgery is one of the options to achieve good cosmetic outcome after a partial mastectomy, it results in donor site scar, pain, and infection as possible postoperative complications. These problems can be reduced by using filling materials, such as oxidized regenerated cellulose (ORC) mesh, which has been reported as a good filling material after partial mastectomy.^[2–6]^ However, in long-term follow-up after the insertion of filling materials, major complications occasionally occur, including surgical site infection or visible deformities. The authors applied a novel technique of combining acellular dermal matrix (ADM) and ORC for partial breast reconstruction and evaluated the cosmetic results after radiotherapy after 2 years of follow-up.

## 2 Methods

This study was approved by the ethics committee of the Chilgok Kyungpook National University Hospital, Daegu, Republic of Korea (2016–04–007). Written informed consent was obtained from all patients before registration, in accordance with the Declaration of Helsinki.

Two patients with breast cancer were enrolled in this study. After partial mastectomies for the removal of breast cancer with clear resection margins, the defects were filled with a combination of ADM (Surgimend; TEI, Biosciences, Inc., Boston, MA) and ORC (Fibrilla; Ethicon, Inc., Johnson & Johnson Company, Somerville, NJ) at the Kyungpook National University Hospital. Neither patient had any underlying disease, such as diabetes or connective tissue disease. Both patients were discharged at 2 days after surgery, and postoperative complications, including seroma formation, occurrence of infection, and postoperative pain, were assessed. The cosmetic results were self-estimated by a survey conducted immediately after surgery and 2 years later according to a 4-point scoring system.^[7,8]^ Clinicopathological factors were also analyzed. Follow-up evaluations were performed using ultrasonography and breast magnetic resonance imaging (MRI).

### 2.1 Surgical techniques

Before surgery, the radiologist performed ultrasound-guided needle localization to identify the main mass and the surrounding, suspicious lesions. These were marked on the breast skin. Patients were laid in the supine position with both arms abducted. After induction of general anesthesia, the needle was inserted, and suspicious lesions were verified again with intraoperative ultrasonography to confirm tumor location. Conventional partial mastectomy was performed with 1- to 2-cm safety margins. Sentinel lymph node biopsies were also performed.

**Figure 1:**
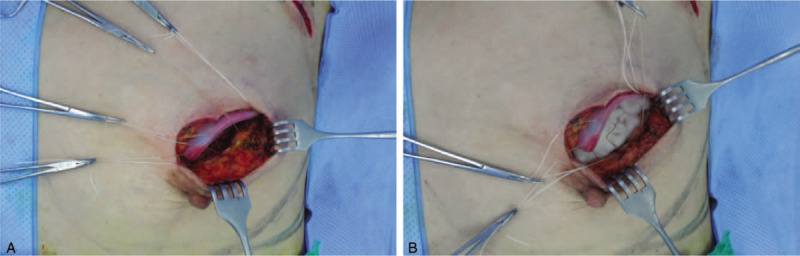
Surgical process using a combination technique with acellular dermal matrix (ADM) and oxidized regenerated cellulose (ORC) after partial mastectomy. (A) Cleaned ADM was fixed to the surgical cavity using white silk 3-0 sutures from the bottom to the side of the surgical cavity. (B) Two ORCs were crumpled and inserted to the surgical cavity to fix the ADM. Finally, the incision was closed using a double-layer skin closure technique.

After partial mastectomy, the specimens were checked by specimen mammography and ultrasonography to confirm that the tumors and suspicious lesions were clearly removed. In addition, intraoperative frozen biopsies from the surgical cavity were performed to ensure negative histologic margins. When the surgical margins were all confirmed as negative, hemostasis and wound irrigation were performed. The defect was then filled with ADM (Surgimend) and 2 ORCs (Fibrilla). First, the surgeon soaked the ADM with normal saline, and dozens of 5-mm pores were inserted using a No. 11 blade. Then, multipored ADM was soaked with povidone-iodine for 5 to 10 minutes. Thereafter, the povidone-iodine-stained ADM was washed out again with normal saline. The washed ADM was fixed to the defect cavity with white silk 3-0 suture at 5 to 7 points to prevent migration of the ADM. Two ORCs were crumpled and inserted inside the ADM (Fig. 1). The surgical incision was closed with a double-layer skin closure using glandular tissue and superficial skin.^[9]^ A closed-suction drain was not inserted to prevent ascending infection. The wounds were closed in 2 layers using interrupted, absorbable 3–0 and 5–0 monofilament sutures (Monosyn; B. Braun Surgical S.A., Barcelona, Spain). A postoperative dressing with an elastic bandage was applied loosely to avoid collapse of the breast.

## 3 Case report

The characteristics of both patients are shown in Table 1.

**Table 1:**
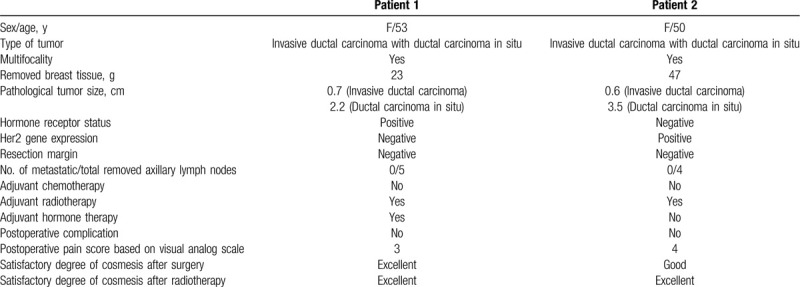
Clinicopathological characteristics of patients with breast cancer who underwent partial mastectomy with a combined acellular dermal matrix and oxidized regenerated cellulose insertion technique.

### 3.1 Patient 1

**Figure 2:**
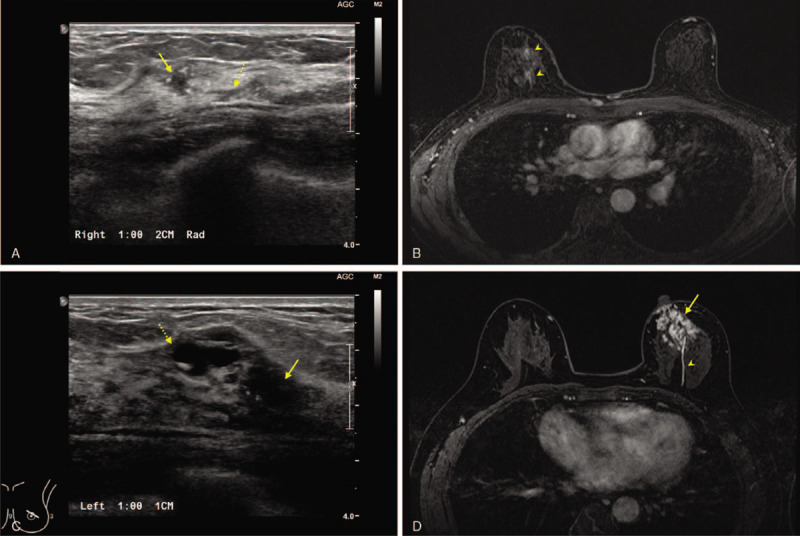
Preoperative findings of the two patients. (A, B) Patient 1 was diagnosed with an invasive ductal carcinoma in her right breast. Ultrasonography revealed a 0.5-cm irregular mass (arrow). Duct dilatation (dotted arrow) was extended at the end of the main lesion. Two different, enhanced lesions (arrowheads) were identified in preoperative breast magnetic resonance imaging (MRI), which correlated with sonographic findings. The total clinical tumor size was 2.6 cm shown on breast MRI. (C, D) Patient 2 was diagnosed with an invasive ductal carcinoma in the periareolar region of her left breast. Ultrasonography detected a 1.2-cm main mass (arrow). A suspicious cystic lesion (dotted arrow) was also identified in the medial side of the main lesion. A total extent of 4.9 cm of the enhanced lump (arrow) was found in the subareolar region of the left breast, and one enhanced duct (arrowhead) from the mass was also verified.

A 53-year-old woman was diagnosed with invasive ductal carcinoma of the right breast by core needle biopsy. Her body mass index was 20.12 kg/m^2^. Breast ultrasound showed an irregular mass with extended ductal dilation with a total extent of approximately 2.6 cm (Fig. 2A). Breast MRI showed that there were multifocal enhancing nodules according to the duct structures (Fig. 2B).

The patient underwent partial mastectomy with sentinel lymph node biopsy. The weight of the removed breast tissue was 33 g. After frozen biopsy revealed all negative findings in the surgical cavity, the defect was filled with ADM and 2 ORCs. On the basis of visual analog pain scores, the mean postoperative pain measured at 3 points during the patient's hospital stay. She was discharged 3 days after surgery. In the final pathological report, the tumor was identified as a 0.7-cm invasive ductal carcinoma with 2.2 cm of ductal carcinoma in situ. The molecular subtype was hormone-positive breast cancer.

**Figure 3:**
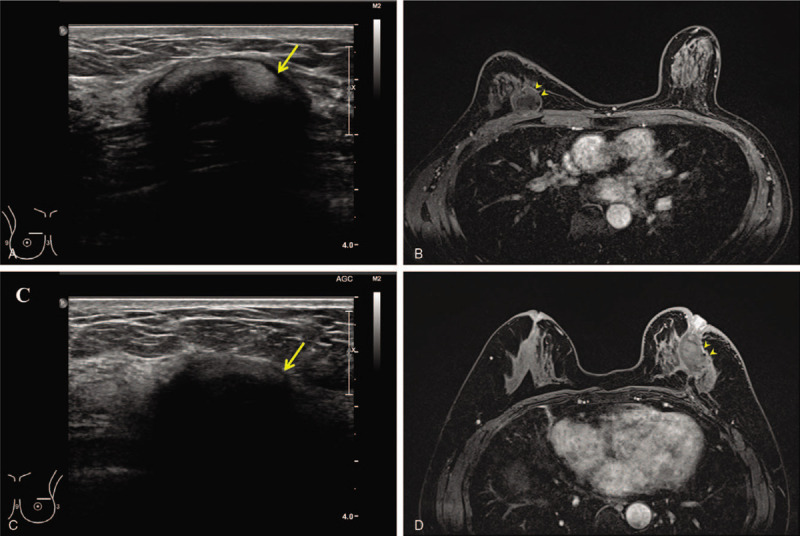
Results of the combination technique of acellular dermal matrix and oxidized regenerated cellulose after partial mastectomy. (A, D) Preoperative views with marking of the breast tumors (dotted circles). (B, E) Immediate postoperative views. Even when the postoperative scars are obviously visible, the breast did not collapse after surgery. (C, F) Postradiation views 1 month after the completion of radiotherapy showing that breast symmetry was well maintained.

**Figure 4:**
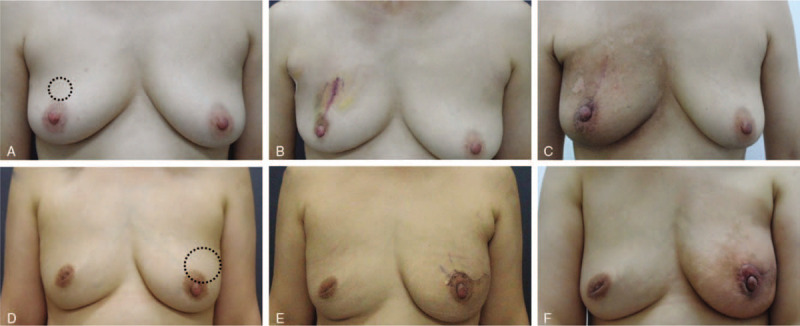
Follow-up imaging of the 2 patients after 2 years. Images were obtained after radiation at 6 months after surgery. (A, B) A complex of acellular dermal matrix (ADM) and oxidized regenerated cellulose (ORC) (arrow) was located on the operative scar. ADM (arrowheads) supported the surgical cavity tightly, and the ORC was almost absorbed and showed a cystic lesion. There was no evidence of any complication or tumor recurrence. (C, D) A complex of ADM and ORC (arrow) was also identified in the outer portion of the left breast. This complex (arrowheads) was located beneath the nipple and supported the nipple to avoid collapse.

The combination of ADM and ORC was formed as a single complex without significant complications shown by ultrasonography and breast MRI at 2 years after surgery (Fig. 3A and B). The patient self-rated the cosmetic result as excellent immediately after surgery and radiotherapy based on the 4-point scoring system (Fig. 4A–C).

### 3.2 Patient 2

A 50-year-old woman was diagnosed with invasive ductal carcinoma of the left breast by core needle biopsy. Her body mass index was 22.86 kg/m^2^. There was a speculated mass with multifocal daughter nodules in breast ultrasound with a total extent of approximately 3.2 cm (Fig. 2C). Breast MRI showed a crumple enhancing mass in the subareolar area and a mass of enhancing duct on the center of the breast (Fig. 2D). The total extent of breast cancer was approximately 4.9 cm as shown on breast MRI. Although the surgeon recommended partial mastectomy with latissimus dorsi (LD) muscle flap for breast reconstruction, the patient refused a flap surgery.

Instead, she underwent partial mastectomy with sentinel lymph node biopsy. The weight of the removed breast tissue was 47 g. After frozen biopsies revealed all negative findings in the surgical cavity, the defect was filled with a combination of ADM and 2 ORCs. On the basis of visual analog pain scores, the patient's mean postoperative pain was at 4 points during her hospital stay. She was discharged 3 days after surgery. The final pathological report noted a tumor measuring 0.6 cm of invasive ductal carcinoma with 3.5 cm of ductal carcinoma in situ. The molecular subtype was HER2-positive breast cancer.

At the 2-year follow-up evaluation, the ADM and ORC were formed as a single complex without major deformity on ultrasonography and breast MRI (Fig. 3C and D). The patient rated the cosmetic result of her breast as good after surgery and excellent after radiotherapy, based on a 4-point scoring system (Fig. 4D–F).

## 4 Discussion

In the present study, ADM was used as a supportive fixture. We confirmed that the cosmetic outcome was well maintained without breast collapse after radiotherapy, even after 2 years. This technique demonstrated several advantages, including short operation time, small incision, and short recovery period. Oncoplastic surgery is aimed at ensuring oncological safety while achieving ideal cosmetic results.^[10]^ Volume displacement means using the remaining breast tissue for breast reconstruction or reduction mammoplasty, which can only be applied when the excised volume is relatively small. Several replacement techniques are used to repair the quadrant defect, including thoracoepigastric flap, lateral thoracodorsal flap, thoracodorsal artery perforator flap, and intercostal artery perforator flap.^[11–15]^ The LD myocutaneous flap and transverse rectus abdominis myocutaneous flap can be used for whole breast reconstruction.^[16–18]^ There are various oncoplastic techniques, but each has limitations and donor site complications.^[19]^

Although the excellent shape of the breast after oncoplastic surgery is an obvious advantage, there are also several disadvantages. Among these are long operation time, high requirements for surgeons, and long postoperative recovery period in volume displacement, especially for the transverse rectus abdominis myocutaneous flap and extended LD flap techniques.^[20,21]^ Many surgeons apply several products that have been proven for their clinical safety to treat partial breast defects. Most studies of oncoplastic surgery have reported good immediate cosmetic results and feasibility as a surgical technique for partial breast defects. However, over the course of long-term follow-up, the cosmetic outcomes worsen and several complications occurred.^[22,23]^

Oncoplastic breast surgery is still a challenge for breast surgeons, and it appears that its possibilities are limitless in terms of surgery. In the past, oncoplastic surgeons were mostly concerned with breast shape regardless of scars. However, more recently, there is an emphasis on not only breast shape but also the length of postoperative scars. To reduce the length of scars, surgeons are attempting to develop filling materials for partial breast defects rather than performing large flap surgeries. However, for use in breast cancer patients, the filling materials must be harmless and oncologically safe. The filling material should also maintain the breast shape and volume, even after radiotherapy. Furthermore, there should be no postoperative recurrence of breast cancer. Because of these demanding requirements, the development of new filling materials is exceedingly difficult. Therefore, many surgeons prefer to use a combination of already available materials that have been proven safe and effective. Several studies have reported filling techniques for partial breast defect using various types of ORCs.^[9,24,25]^ However, in the long-term follow-up, many of these cases demonstrated poor cosmetic results, usually because of breast collapse. These results could be because of the absence of a supportive structure. Therefore, in this study, we used ADM as a supportive structure to prevent breast collapse.

## 5 Conclusions

In our study using ADM combined with ORC, we found that this method was oncologically safe, there was no recurrence of breast cancer, the breast shape was well maintained, and there were no major complications reported. The use of ADM combined with ORC for partial defects in patients with breast cancer is a feasible option with good cosmetic outcomes. This technique can also be applied for breast cancer patients without major deformity.

## Author contributions

**Acquisition of data:** Jeeyeon Lee and Jeong Woo Lee.

**Analysis and interpretation:** Joon Seok Lee and Junjie Li.

**Conceptualization:** Jeeyeon Lee, Ho Yong Park.

**Data curation:** Jung Dug Yang, Jeong Woo Lee, Junjie Li, Jin Hyang Jung, Joon Seok Lee.

**Formal analysis:** Junjie Li, Jin Hyang Jung, Wan Wook Kim, Joon Seok Lee.

**Investigation:** Jung Dug Yang, Jeong Woo Lee, Junjie Li.

**Methodology:** Jung Dug Yang, Jin Hyang Jung.

**Study concept and design:** Ho Yong Park and Jung Dug Yang

**Study supervision:** Jin Hyang Jung and Wan Wook Kim

**Supervision:** Jung Dug Yang, Jin Hyang Jung, Wan Wook Kim, Chan Sub Park.

**Writing – original draft:** Jeeyeon Lee.

**Writing – review & editing:** Jeeyeon Lee.
